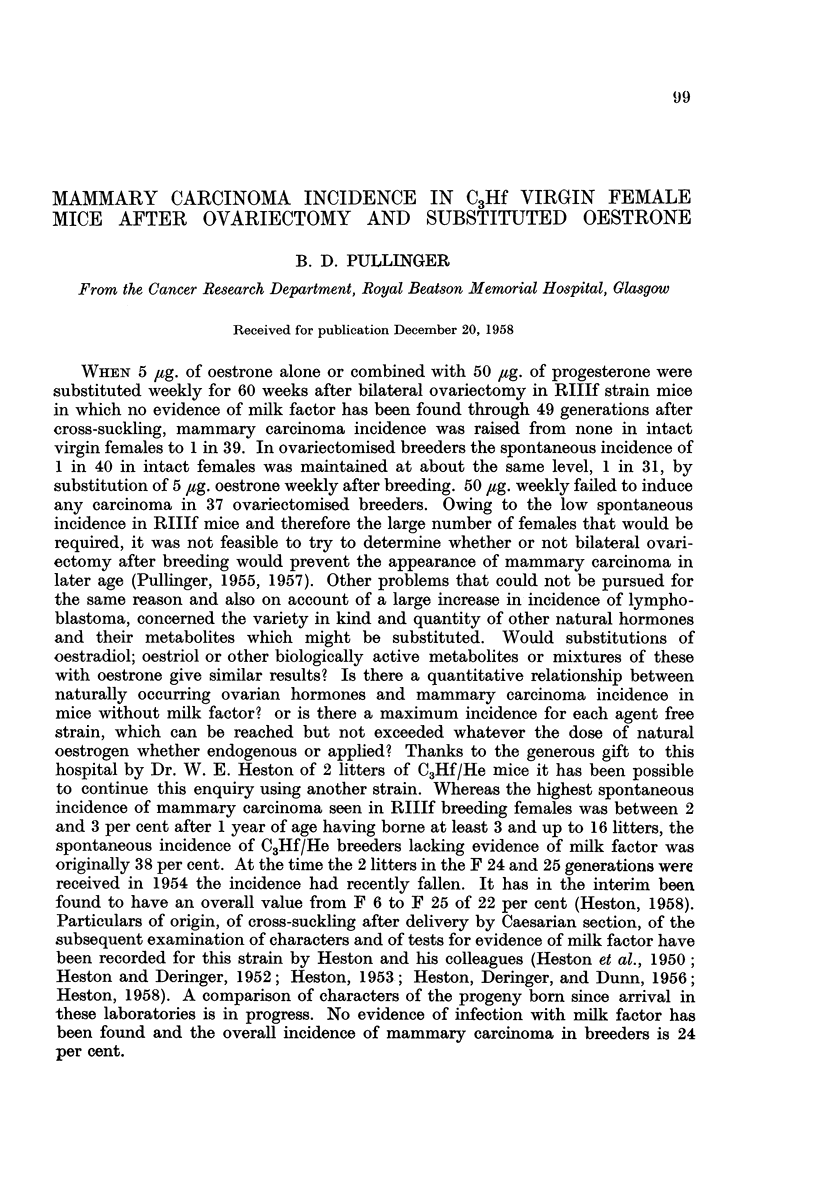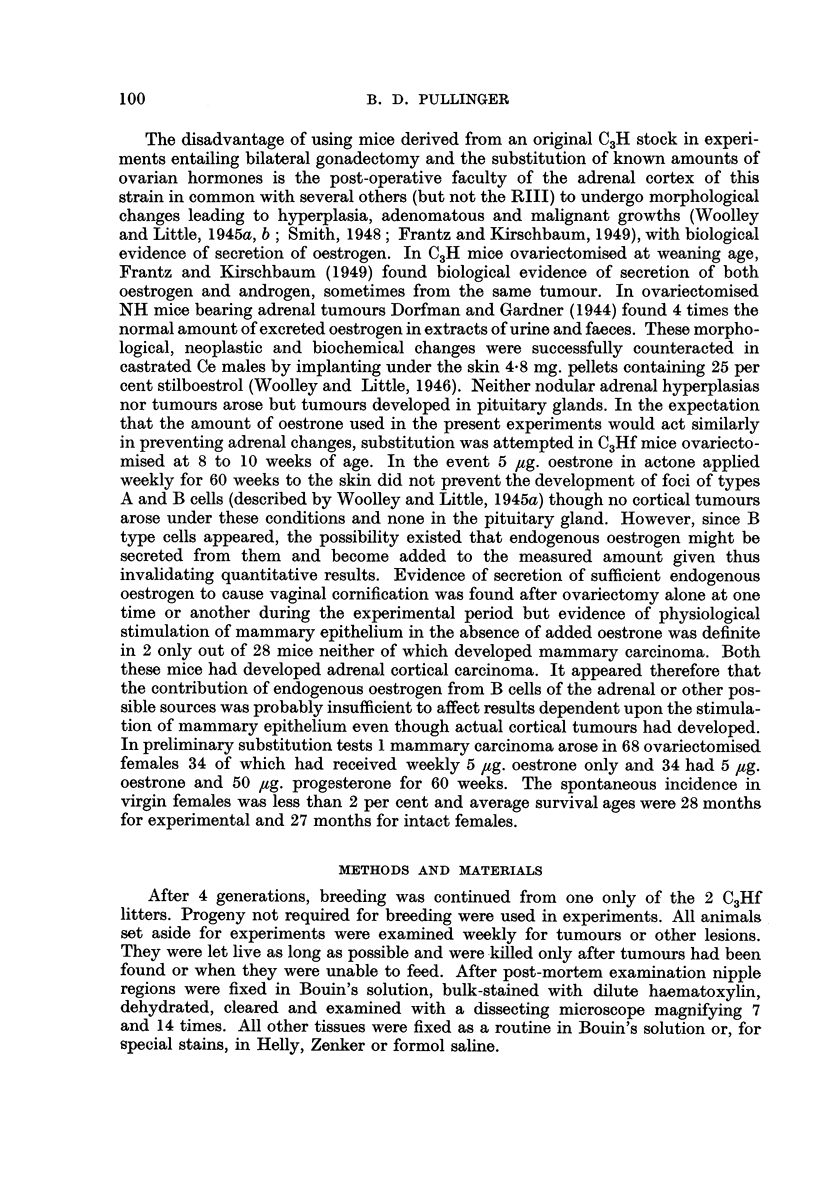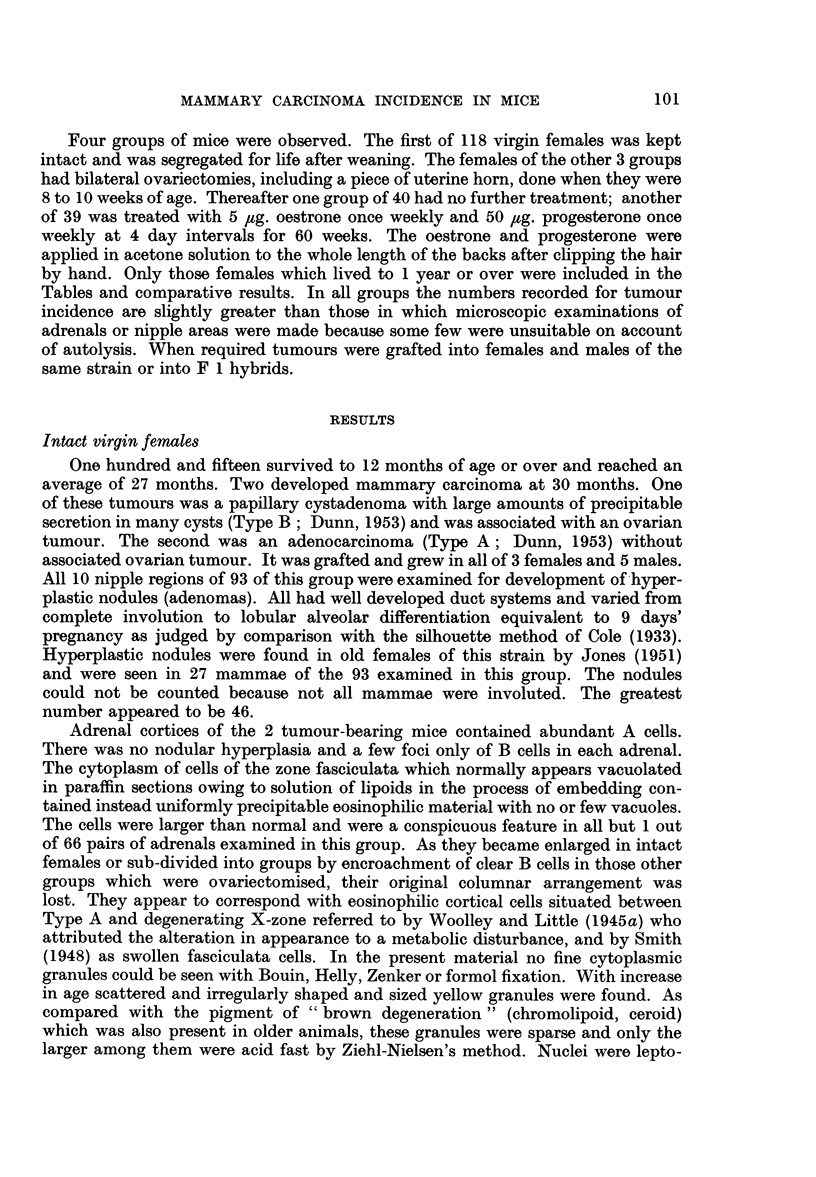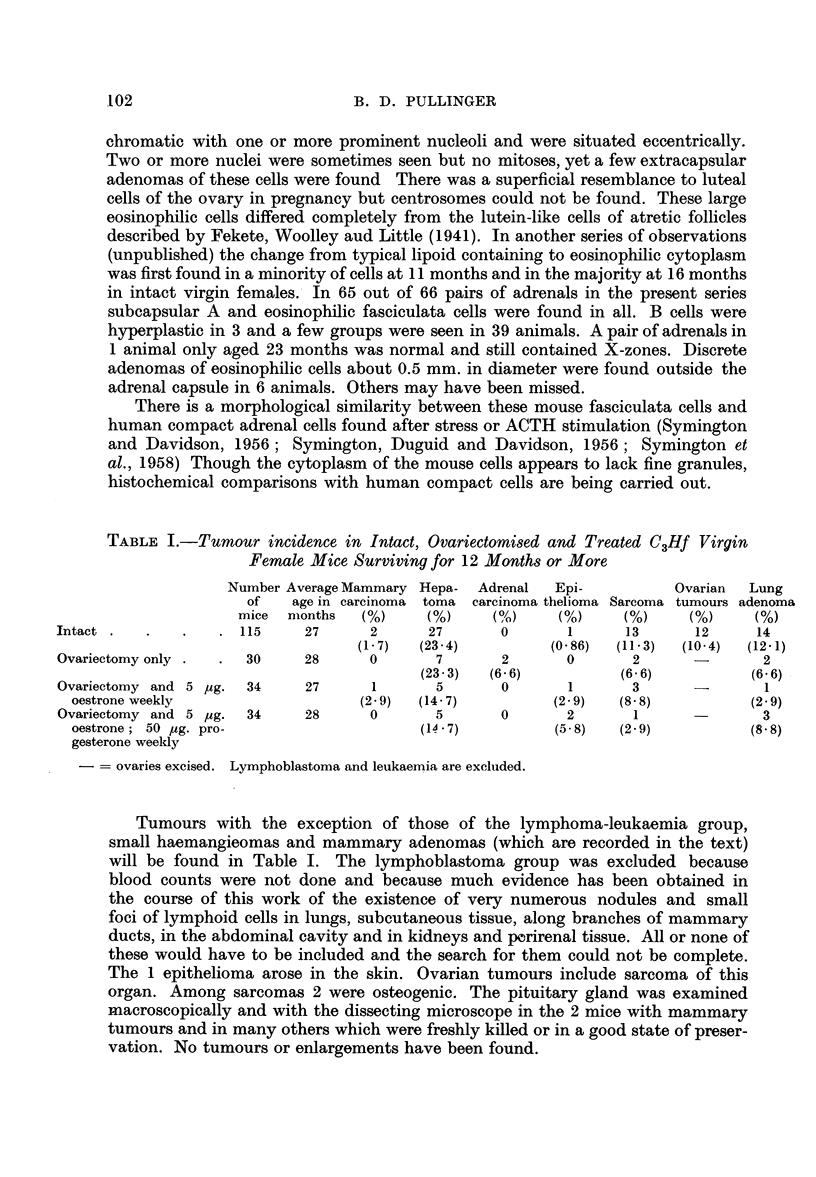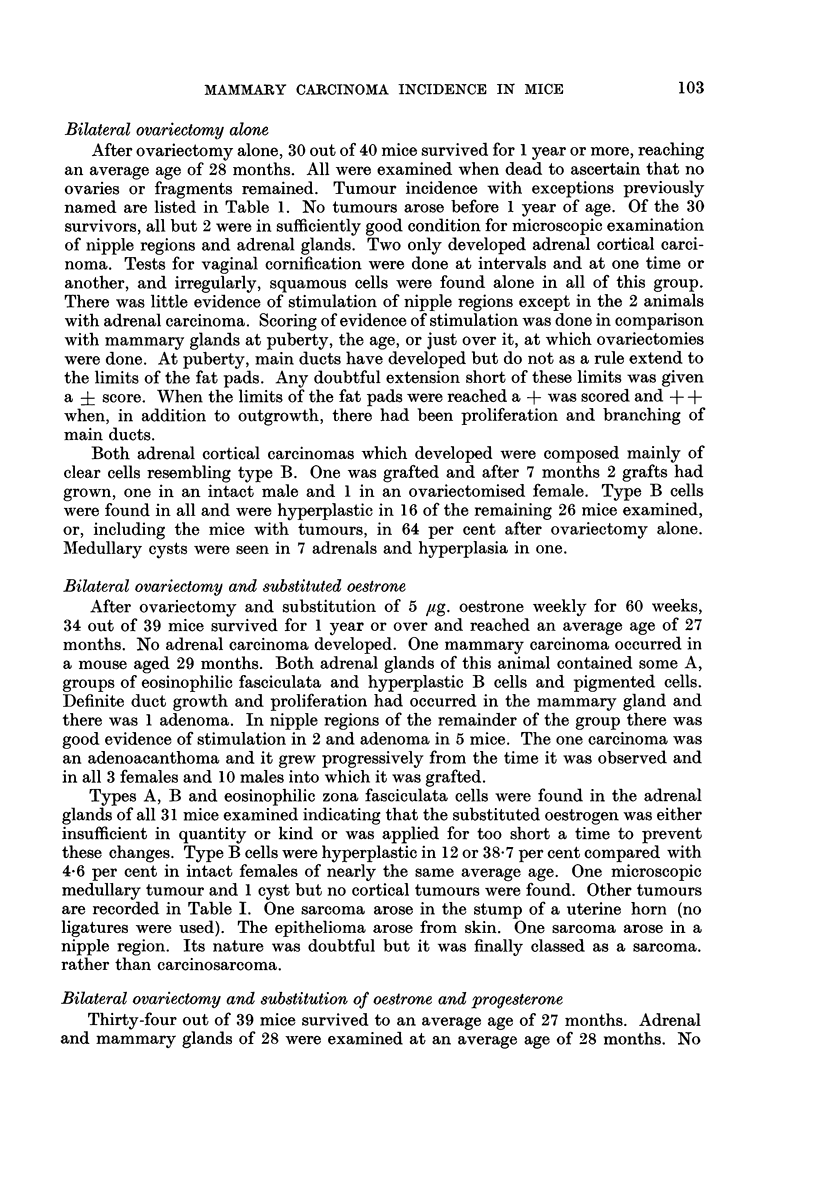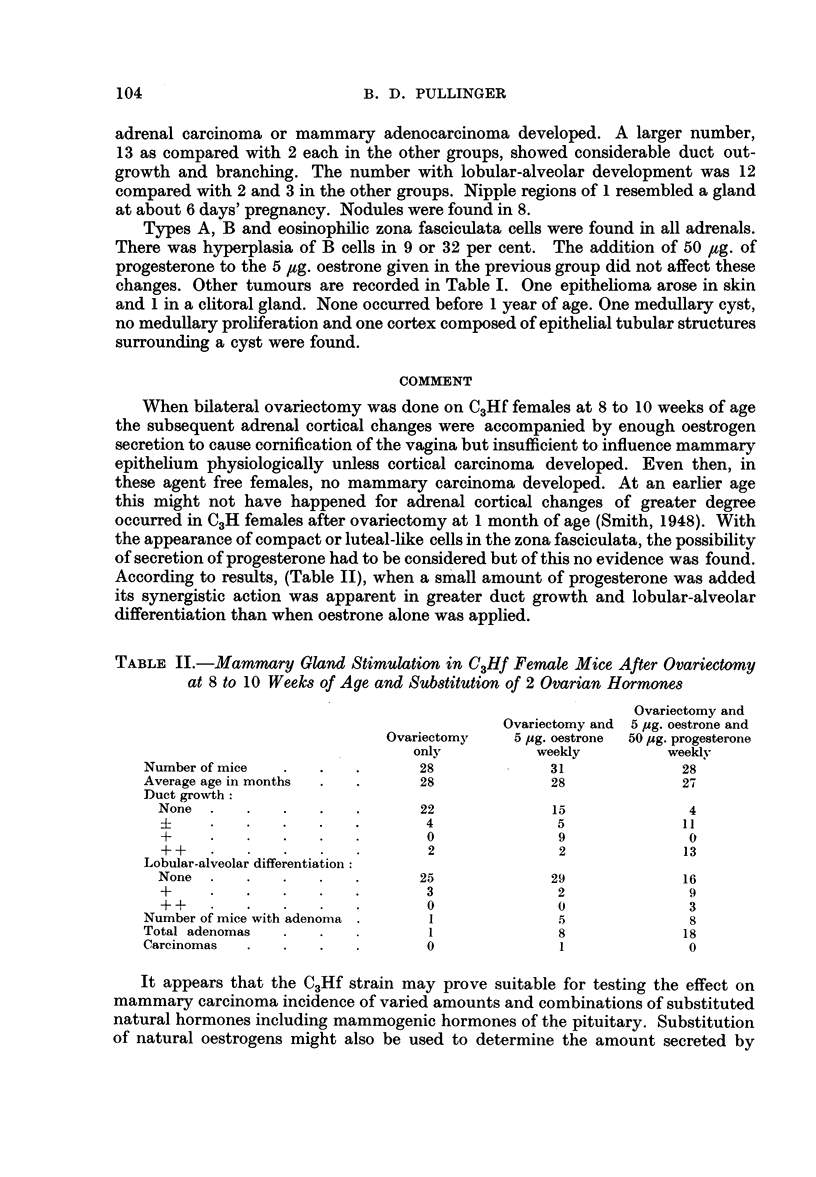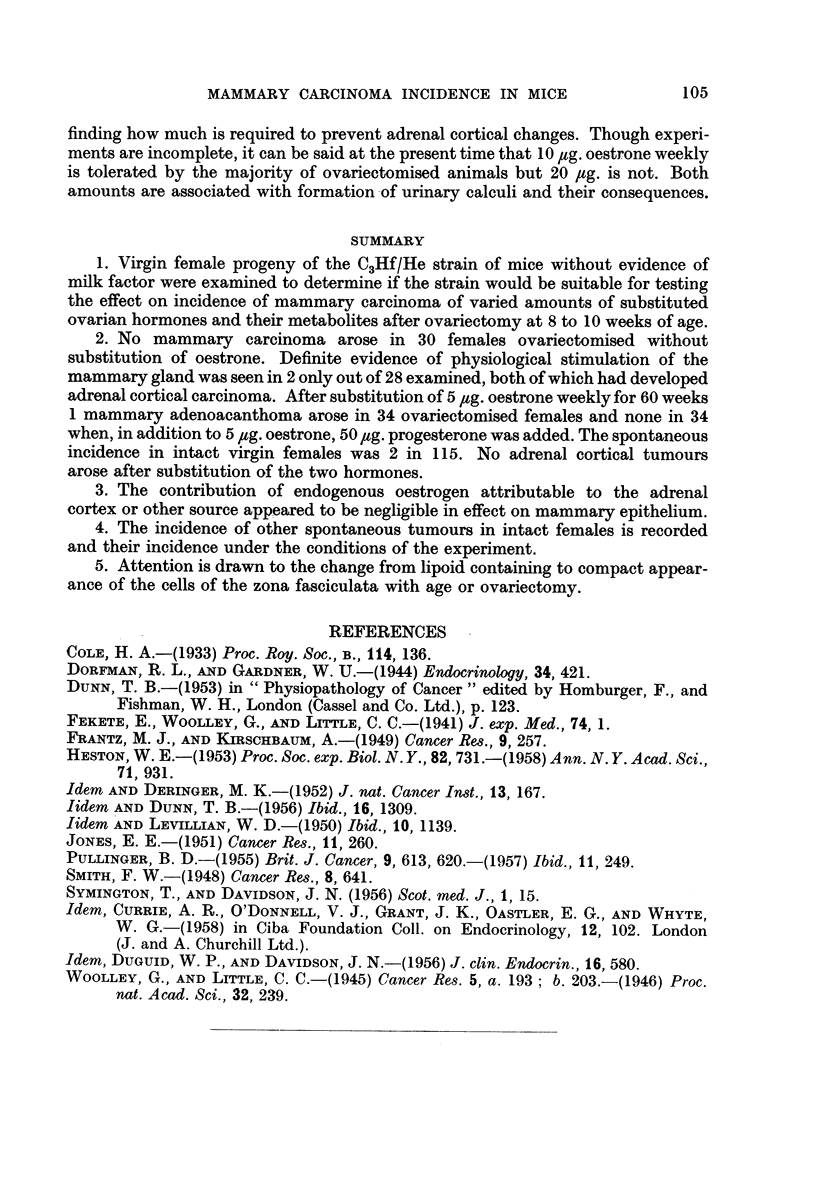# Mammary Carcinoma Incidence in C_3_Hf Virgin Female Mice after Ovariectomy and Substituted Oestrone

**DOI:** 10.1038/bjc.1959.14

**Published:** 1959-03

**Authors:** B. D. Pullinger


### MAMMARY CARCINOMA INCIDENCE IN $C_3Hf$ VIRGIN FEMALE MICE AFTER OVARIECTOMY AND SUBSTITUTED OESTRONE

B. D. PULLINGER

*From the Cancer Research Department, Royal Beatson Memorial Hospital, Glasgow*

Received for publication December 20, 1958

WHEN 5 $\mu g$. of oestrone alone or combined with 50 $\mu g$. of progesterone were substituted weekly for 60 weeks after bilateral ovariectomy in RIIIf strain mice in which no evidence of milk factor has been found through 49 generations after cross-suckling, mammary carcinoma incidence was raised from none in intact virgin females to 1 in 39. In ovariectomised breeders the spontaneous incidence of 1 in 40 in intact females was maintained at about the same level, 1 in 31, by substitution of 5 $\mu g$. oestrone weekly after breeding. 50 $\mu g$. weekly failed to induce any carcinoma in 37 ovariectomised breeders. Owing to the low spontaneous incidence in RIIIf mice and therefore the large number of females that would be required, it was not feasible to try to determine whether or not bilateral ovariectomy after breeding would prevent the appearance of mammary carcinoma in later age (Pullinger, 1955, 1957). Other problems that could not be pursued for the same reason and also on account of a large increase in incidence of lymphoblastoma, concerned the variety in kind and quantity of other natural hormones and their metabolites which might be substituted. Would substitutions of oestradiol; oestriol or other biologically active metabolites or mixtures of these with oestrone give similar results? Is there a quantitative relationship between naturally occurring ovarian hormones and mammary carcinoma incidence in mice without milk factor? or is there a maximum incidence for each agent free strain, which can be reached but not exceeded whatever the dose of natural oestrogen whether endogenous or applied? Thanks to the generous gift to this hospital by Dr. W. E. Heston of 2 litters of $C_3Hf/He$ mice it has been possible to continue this enquiry using another strain. Whereas the highest spontaneous incidence of mammary carcinoma seen in RIIIf breeding females was between 2 and 3 per cent after 1 year of age having borne at least 3 and up to 16 litters, the spontaneous incidence of $C_3Hf/He$ breeders lacking evidence of milk factor was originally 38 per cent. At the time the 2 litters in the F 24 and 25 generations were received in 1954 the incidence had recently fallen. It has in the interim been found to have an overall value from F 6 to F 25 of 22 per cent (Heston, 1958). Particulars of origin, of cross-suckling after delivery by Caesarian section, of the subsequent examination of characters and of tests for evidence of milk factor have been recorded for this strain by Heston and his colleagues (Heston *et al.*, 1950; Heston and Deringer, 1952; Heston, 1953; Heston, Deringer, and Dunn, 1956; Heston, 1958). A comparison of characters of the progeny born since arrival in these laboratories is in progress. No evidence of infection with milk factor has been found and the overall incidence of mammary carcinoma in breeders is 24 per cent.

The disadvantage of using mice derived from an original $C_3H$ stock in experiments entailing bilateral gonadectomy and the substitution of known amounts of ovarian hormones is the post-operative faculty of the adrenal cortex of this strain in common with several others (but not the RIII) to undergo morphological changes leading to hyperplasia, adenomatous and malignant growths (Woolley and Little, 1945*a*, *b* ; Smith, 1948 ; Frantz and Kirschbaum, 1949), with biological evidence of secretion of oestrogen. In $C_3H$ mice ovariectomised at weaning age, Frantz and Kirschbaum (1949) found biological evidence of secretion of both oestrogen and androgen, sometimes from the same tumour. In ovariectomised NH mice bearing adrenal tumours Dorfman and Gardner (1944) found 4 times the normal amount of excreted oestrogen in extracts of urine and faeces. These morphological, neoplastic and biochemical changes were successfully counteracted in castrated Ce males by implanting under the skin 4·8 mg. pellets containing 25 per cent stilboestrol (Woolley and Little, 1946). Neither nodular adrenal hyperplasias nor tumours arose but tumours developed in pituitary glands. In the expectation that the amount of oestrone used in the present experiments would act similarly in preventing adrenal changes, substitution was attempted in $C_3Hf$ mice ovariectomised at 8 to 10 weeks of age. In the event 5 $\mu$g. oestrone in actone applied weekly for 60 weeks to the skin did not prevent the development of foci of types A and B cells (described by Woolley and Little, 1945*a*) though no cortical tumours arose under these conditions and none in the pituitary gland. However, since B type cells appeared, the possibility existed that endogenous oestrogen might be secreted from them and become added to the measured amount given thus invalidating quantitative results. Evidence of secretion of sufficient endogenous oestrogen to cause vaginal cornification was found after ovariectomy alone at one time or another during the experimental period but evidence of physiological stimulation of mammary epithelium in the absence of added oestrone was definite in 2 only out of 28 mice neither of which developed mammary carcinoma. Both these mice had developed adrenal cortical carcinoma. It appeared therefore that the contribution of endogenous oestrogen from B cells of the adrenal or other possible sources was probably insufficient to affect results dependent upon the stimulation of mammary epithelium even though actual cortical tumours had developed. In preliminary substitution tests 1 mammary carcinoma arose in 68 ovariectomised females 34 of which had received weekly 5 $\mu$g. oestrone only and 34 had 5 $\mu$g. oestrone and 50 $\mu$g. progesterone for 60 weeks. The spontaneous incidence in virgin females was less than 2 per cent and average survival ages were 28 months for experimental and 27 months for intact females.

### METHODS AND MATERIALS

After 4 generations, breeding was continued from one only of the 2 $C_3Hf$ litters. Progeny not required for breeding were used in experiments. All animals set aside for experiments were examined weekly for tumours or other lesions. They were let live as long as possible and were killed only after tumours had been found or when they were unable to feed. After post-mortem examination nipple regions were fixed in Bouin's solution, bulk-stained with dilute haematoxylin, dehydrated, cleared and examined with a dissecting microscope magnifying 7 and 14 times. All other tissues were fixed as a routine in Bouin's solution or, for special stains, in Helly, Zenker or formol saline.

Four groups of mice were observed. The first of 118 virgin females was kept intact and was segregated for life after weaning. The females of the other 3 groups had bilateral ovariectomies, including a piece of uterine horn, done when they were 8 to 10 weeks of age. Thereafter one group of 40 had no further treatment; another of 39 was treated with 5 $\mu$g. oestrone once weekly and 50 $\mu$g. progesterone once weekly at 4 day intervals for 60 weeks. The oestrone and progesterone were applied in acetone solution to the whole length of the backs after clipping the hair by hand. Only those females which lived to 1 year or over were included in the Tables and comparative results. In all groups the numbers recorded for tumour incidence are slightly greater than those in which microscopic examinations of adrenals or nipple areas were made because some few were unsuitable on account of autolysis. When required tumours were grafted into females and males of the same strain or into F 1 hybrids.

### RESULTS

### *Intact virgin females*

One hundred and fifteen survived to 12 months of age or over and reached an average of 27 months. Two developed mammary carcinoma at 30 months. One of these tumours was a papillary cystadenoma with large amounts of precipitable secretion in many cysts (Type B ; Dunn, 1953) and was associated with an ovarian tumour. The second was an adenocarcinoma (Type A ; Dunn, 1953) without associated ovarian tumour. It was grafted and grew in all of 3 females and 5 males. All 10 nipple regions of 93 of this group were examined for development of hyperplastic nodules (adenomas). All had well developed duct systems and varied from complete involution to lobular alveolar differentiation equivalent to 9 days' pregnancy as judged by comparison with the silhouette method of Cole (1933). Hyperplastic nodules were found in old females of this strain by Jones (1951) and were seen in 27 mammae of the 93 examined in this group. The nodules could not be counted because not all mammae were involuted. The greatest number appeared to be 46.

Adrenal cortices of the 2 tumour-bearing mice contained abundant A cells. There was no nodular hyperplasia and a few foci only of B cells in each adrenal. The cytoplasm of cells of the zone fasciculata which normally appears vacuolated in paraffin sections owing to solution of lipoids in the process of embedding contained instead uniformly precipitable eosinophilic material with no or few vacuoles. The cells were larger than normal and were a conspicuous feature in all but 1 out of 66 pairs of adrenals examined in this group. As they became enlarged in intact females or sub-divided into groups by encroachment of clear B cells in those other groups which were ovariectomised, their original columnar arrangement was lost. They appear to correspond with eosinophilic cortical cells situated between Type A and degenerating X-zone referred to by Woolley and Little (1945*a*) who attributed the alteration in appearance to a metabolic disturbance, and by Smith (1948) as swollen fasciculata cells. In the present material no fine cytoplasmic granules could be seen with Bouin, Helly, Zenker or formol fixation. With increase in age scattered and irregularly shaped and sized yellow granules were found. As compared with the pigment of " brown degeneration " (chromolipoid, ceroid) which was also present in older animals, these granules were sparse and only the larger among them were acid fast by Ziehl-Nielsen's method. Nuclei were leptochromatic with one or more prominent nucleoli and were situated eccentrically. Two or more nuclei were sometimes seen but no mitoses, yet a few extracapsular adenomas of these cells were found There was a superficial resemblance to luteal cells of the ovary in pregnancy but centrosomes could not be found. These large eosinophilic cells differed completely from the lutein-like cells of atretic follicles described by Fekete, Woolley aud Little (1941). In another series of observations (unpublished) the change from typical lipoid containing to eosinophilic cytoplasm was first found in a minority of cells at 11 months and in the majority at 16 months in intact virgin females. In 65 out of 66 pairs of adrenals in the present series subcapsular A and eosinophilic fasciculata cells were found in all. B cells were hyperplastic in 3 and a few groups were seen in 39 animals. A pair of adrenals in 1 animal only aged 23 months was normal and still contained X-zones. Discrete adenomas of eosinophilic cells about 0.5 mm. in diameter were found outside the adrenal capsule in 6 animals. Others may have been missed.

There is a morphological similarity between these mouse fasciculata cells and human compact adrenal cells found after stress or ACTH stimulation (Symington and Davidson, 1956 ; Symington, Duguid and Davidson, 1956 ; Symington *et al.*, 1958) Though the cytoplasm of the mouse cells appears to lack fine granules, histochemical comparisons with human compact cells are being carried out.

TABLE I.—*Tumour incidence in Intact, Ovariectomised and Treated $C_3Hf$ Virgin Female Mice Surviving for* 12 *Months or More*

| | Number of mice | Average age in months | Mammary carcinoma (%) | Hepatoma (%) | Adrenal carcinoma (%) | Epithelioma (%) | Sarcoma (%) | Ovarian tumours (%) | Lung adenoma (%) |
|---|---|---|---|---|---|---|---|---|---|
| Intact . . . . | 115 | 27 | 2 (1·7) | 27 (23·4) | 0 | 1 (0·86) | 13 (11·3) | 12 (10·4) | 14 (12·1) |
| Ovariectomy only . . | 30 | 28 | 0 | 7 (23·3) | 2 (6·6) | 0 | 2 (6·6) | — | 2 (6·6) |
| Ovariectomy and 5 μg. oestrone weekly | 34 | 27 | 1 (2·9) | 5 (14·7) | 0 | 1 (2·9) | 3 (8·8) | — | 1 (2·9) |
| Ovariectomy and 5 μg. oestrone ; 50 μg. progesterone weekly | 34 | 28 | 0 | 5 (14·7) | 0 | 2 (5·8) | 1 (2·9) | — | 3 (8·8) |

— = ovaries excised. Lymphoblastoma and leukaemia are excluded.

Tumours with the exception of those of the lymphoma-leukaemia group, small haemangieomas and mammary adenomas (which are recorded in the text) will be found in Table I. The lymphoblastoma group was excluded because blood counts were not done and because much evidence has been obtained in the course of this work of the existence of very numerous nodules and small foci of lymphoid cells in lungs, subcutaneous tissue, along branches of mammary ducts, in the abdominal cavity and in kidneys and perirenal tissue. All or none of these would have to be included and the search for them could not be complete. The 1 epithelioma arose in the skin. Ovarian tumours include sarcoma of this organ. Among sarcomas 2 were osteogenic. The pituitary gland was examined macroscopically and with the dissecting microscope in the 2 mice with mammary tumours and in many others which were freshly killed or in a good state of preservation. No tumours or enlargements have been found.

*Bilateral ovariectomy alone*

After ovariectomy alone, 30 out of 40 mice survived for 1 year or more, reaching an average age of 28 months. All were examined when dead to ascertain that no ovaries or fragments remained. Tumour incidence with exceptions previously named are listed in Table 1. No tumours arose before 1 year of age. Of the 30 survivors, all but 2 were in sufficiently good condition for microscopic examination of nipple regions and adrenal glands. Two only developed adrenal cortical carcinoma. Tests for vaginal cornification were done at intervals and at one time or another, and irregularly, squamous cells were found alone in all of this group. There was little evidence of stimulation of nipple regions except in the 2 animals with adrenal carcinoma. Scoring of evidence of stimulation was done in comparison with mammary glands at puberty, the age, or just over it, at which ovariectomies were done. At puberty, main ducts have developed but do not as a rule extend to the limits of the fat pads. Any doubtful extension short of these limits was given a $\pm$ score. When the limits of the fat pads were reached a $+$ was scored and $++$ when, in addition to outgrowth, there had been proliferation and branching of main ducts.

Both adrenal cortical carcinomas which developed were composed mainly of clear cells resembling type B. One was grafted and after 7 months 2 grafts had grown, one in an intact male and 1 in an ovariectomised female. Type B cells were found in all and were hyperplastic in 16 of the remaining 26 mice examined, or, including the mice with tumours, in 64 per cent after ovariectomy alone. Medullary cysts were seen in 7 adrenals and hyperplasia in one.

*Bilateral ovariectomy and substituted oestrone*

After ovariectomy and substitution of 5 $\mu$g. oestrone weekly for 60 weeks, 34 out of 39 mice survived for 1 year or over and reached an average age of 27 months. No adrenal carcinoma developed. One mammary carcinoma occurred in a mouse aged 29 months. Both adrenal glands of this animal contained some A, groups of eosinophilic fasciculata and hyperplastic B cells and pigmented cells. Definite duct growth and proliferation had occurred in the mammary gland and there was 1 adenoma. In nipple regions of the remainder of the group there was good evidence of stimulation in 2 and adenoma in 5 mice. The one carcinoma was an adenoacanthoma and it grew progressively from the time it was observed and in all 3 females and 10 males into which it was grafted.

Types A, B and eosinophilic zona fasciculata cells were found in the adrenal glands of all 31 mice examined indicating that the substituted oestrogen was either insufficient in quantity or kind or was applied for too short a time to prevent these changes. Type B cells were hyperplastic in 12 or 38·7 per cent compared with 4·6 per cent in intact females of nearly the same average age. One microscopic medullary tumour and 1 cyst but no cortical tumours were found. Other tumours are recorded in Table I. One sarcoma arose in the stump of a uterine horn (no ligatures were used). The epithelioma arose from skin. One sarcoma arose in a nipple region. Its nature was doubtful but it was finally classed as a sarcoma. rather than carcinosarcoma.

*Bilateral ovariectomy and substitution of oestrone and progesterone*

Thirty-four out of 39 mice survived to an average age of 27 months. Adrenal and mammary glands of 28 were examined at an average age of 28 months. No adrenal carcinoma or mammary adenocarcinoma developed. A larger number, 13 as compared with 2 each in the other groups, showed considerable duct outgrowth and branching. The number with lobular-alveolar development was 12 compared with 2 and 3 in the other groups. Nipple regions of 1 resembled a gland at about 6 days' pregnancy. Nodules were found in 8.

Types A, B and eosinophilic zona fasciculata cells were found in all adrenals. There was hyperplasia of B cells in 9 or 32 per cent. The addition of 50 $\mu$g. of progesterone to the 5 $\mu$g. oestrone given in the previous group did not affect these changes. Other tumours are recorded in Table I. One epithelioma arose in skin and 1 in a clitoral gland. None occurred before 1 year of age. One medullary cyst, no medullary proliferation and one cortex composed of epithelial tubular structures surrounding a cyst were found.

### COMMENT

When bilateral ovariectomy was done on $C_3$Hf females at 8 to 10 weeks of age the subsequent adrenal cortical changes were accompanied by enough oestrogen secretion to cause cornification of the vagina but insufficient to influence mammary epithelium physiologically unless cortical carcinoma developed. Even then, in these agent free females, no mammary carcinoma developed. At an earlier age this might not have happened for adrenal cortical changes of greater degree occurred in $C_3$H females after ovariectomy at 1 month of age (Smith, 1948). With the appearance of compact or luteal-like cells in the zona fasciculata, the possibility of secretion of progesterone had to be considered but of this no evidence was found. According to results, (Table II), when a small amount of progesterone was added its synergistic action was apparent in greater duct growth and lobular-alveolar differentiation than when oestrone alone was applied.

TABLE II.—*Mammary Gland Stimulation in $C_3$Hf Female Mice After Ovariectomy at 8 to 10 Weeks of Age and Substitution of 2 Ovarian Hormones*

| | Ovariectomy only | Ovariectomy and 5 $\mu$g. oestrone weekly | Ovariectomy and 5 $\mu$g. oestrone and 50 $\mu$g. progesterone weekly |
|---|---|---|---|
| Number of mice | 28 | 31 | 28 |
| Average age in months | 28 | 28 | 27 |
| Duct growth : | | | |
| None | 22 | 15 | 4 |
| ± | 4 | 5 | 11 |
| + | 0 | 9 | 0 |
| ++ | 2 | 2 | 13 |
| Lobular-alveolar differentiation : | | | |
| None | 25 | 29 | 16 |
| + | 3 | 2 | 9 |
| ++ | 0 | 0 | 3 |
| Number of mice with adenoma | 1 | 5 | 8 |
| Total adenomas | 1 | 8 | 18 |
| Carcinomas | 0 | 1 | 0 |

It appears that the $C_3$Hf strain may prove suitable for testing the effect on mammary carcinoma incidence of varied amounts and combinations of substituted natural hormones including mammogenic hormones of the pituitary. Substitution of natural oestrogens might also be used to determine the amount secreted by finding how much is required to prevent adrenal cortical changes. Though experiments are incomplete, it can be said at the present time that 10 $\mu$g. oestrone weekly is tolerated by the majority of ovariectomised animals but 20 $\mu$g. is not. Both amounts are associated with formation of urinary calculi and their consequences.

### SUMMARY

1. Virgin female progeny of the $C_3$Hf/He strain of mice without evidence of milk factor were examined to determine if the strain would be suitable for testing the effect on incidence of mammary carcinoma of varied amounts of substituted ovarian hormones and their metabolites after ovariectomy at 8 to 10 weeks of age.

2. No mammary carcinoma arose in 30 females ovariectomised without substitution of oestrone. Definite evidence of physiological stimulation of the mammary gland was seen in 2 only out of 28 examined, both of which had developed adrenal cortical carcinoma. After substitution of 5 $\mu$g. oestrone weekly for 60 weeks 1 mammary adenoacanthoma arose in 34 ovariectomised females and none in 34 when, in addition to 5 $\mu$g. oestrone, 50 $\mu$g. progesterone was added. The spontaneous incidence in intact virgin females was 2 in 115. No adrenal cortical tumours arose after substitution of the two hormones.

3. The contribution of endogenous oestrogen attributable to the adrenal cortex or other source appeared to be negligible in effect on mammary epithelium.

4. The incidence of other spontaneous tumours in intact females is recorded and their incidence under the conditions of the experiment.

5. Attention is drawn to the change from lipoid containing to compact appearance of the cells of the zona fasciculata with age or ovariectomy.

### REFERENCES

Cole, H. A.—(1933) *Proc. Roy. Soc.*, b., **114**, 136.

Dorfman, R. L., and Gardner, W. U.—(1944) *Endocrinology*, **34**, 421.

Dunn, T. B.—(1953) in " Physiopathology of Cancer " edited by Homburger, F., and Fishman, W. H., London (Cassel and Co. Ltd.), p. 123.

Fekete, E., Woolley, G., and Little, C. C.—(1941) *J. exp. Med.*, **74**, 1.

Frantz, M. J., and Kirschbaum, A.—(1949) *Cancer Res.*, **9**, 257.

Heston, W. E.—(1953) *Proc. Soc. exp. Biol. N.Y.*, **82**, 731.—(1958) *Ann. N.Y. Acad. Sci.*, **71**, 931.

*Idem* and Deringer, M. K.—(1952) *J. nat. Cancer Inst.*, **13**, 167.

*Iidem* and Dunn, T. B.—(1956) *Ibid.*, **16**, 1309.

*Iidem* and Levillian, W. D.—(1950) *Ibid.*, **10**, 1139.

Jones, E. E.—(1951) *Cancer Res.*, **11**, 260.

Pullinger, B. D.—(1955) *Brit. J. Cancer*, **9**, 613, 620.—(1957) *Ibid.*, **11**, 249.

Smith, F. W.—(1948) *Cancer Res.*, **8**, 641.

Symington, T., and Davidson, J. N. (1956) *Scot. med. J.*, **1**, 15.

*Idem*, Currie, A. R., O'Donnell, V. J., Grant, J. K., Oastler, E. G., and Whyte, W. G.—(1958) in Ciba Foundation Coll. on Endocrinology, **12**, 102. London (J. and A. Churchill Ltd.).

*Idem*, Duguid, W. P., and Davidson, J. N.—(1956) *J. clin. Endocrin.*, **16**, 580.

Woolley, G., and Little, C. C.—(1945) *Cancer Res.* **5**, *a.* 193 ; *b.* 203.—(1946) *Proc. nat. Acad. Sci.*, **32**, 239.